# A novel liquid-liquid phase separation related gene signature including ARL6IP4 predicts prognosis and immune landscape in colorectal cancer

**DOI:** 10.3389/fimmu.2025.1694377

**Published:** 2026-01-06

**Authors:** Tao Jiang, Yuqiu Li, Ning Ma, Junnan Chen, Hu Song, Yixin Xu, Jun Song

**Affiliations:** 1 Department of General Surgery, The Affiliated Hospital of Xuzhou Medical University, Xuzhou, Jiangsu, China; 2 Institute of Digestive Diseases, Xuzhou Medical University, Xuzhou, Jiangsu, China; 3 Affiliated First Clinical College, Xuzhou Medical University, Xuzhou, Jiangsu, China; 4 Central Laboratory, The Affiliated Hospital of Xuzhou Medical University, Xuzhou, Jiangsu, China

**Keywords:** ARL6IP4, colorectal cancer, liquid-liquid phase separation, prognosis, tumor microenvironment

## Abstract

**Background:**

Colorectal cancer (CRC) ranks as the second leading cause of cancer-related mortality worldwide. Liquid-liquid phase separation (LLPS) is a phenomenon driven by multivalent weak interactions among biomolecules, such as proteins and nucleic acids, which results in the formation of biomolecular condensates. Abnormal LLPS is closely linked to tumorigenesis and progression. However, a prognostic signature based on LLPS-related genes (LRGs) has rarely been established in CRC.

**Methods:**

We retrieved CRC data from The Cancer Genome Atlas (TCGA) and Gene Expression Omnibus (GEO) databases. LRGs were obtained from the DrLLPS database. A novel signature was developed in the TCGA training cohort using univariate Cox regression, least absolute shrinkage and selection operator (LASSO) Cox regression and multivariate Cox regression. Subsequently, a risk signature termed the LLPS-related risk score (LRS) was introduced, categorizing CRC patients into high-risk and low-risk groups based on their LRS. Furthermore, the efficacy of the risk signature was evaluated using principal component analysis (PCA), Kaplan-Meier survival, receiver operating characteristic (ROC), nomogram, and concordance index (C-index). Additionally, the differences in immune cell infiltration, immune function, the tumor microenvironment (TME), drug sensitivity, and tumor mutation burden (TMB) between these risk subgroups were analyzed. Importantly, experimental investigations were conducted to assess the expression levels and LLPS capabilities of ARF Like GTPase 6 Interacting Protein 4 (ARL6IP4)in CRC.

**Results:**

We developed an eight-LRG prognostic risk signature based on LRGs and confirmed its role as an independent prognostic factor for patients with CRC. Additionally, we observed significant differences in immune cell infiltration, immune function, and TME between the low-risk and high-risk groups. Furthermore, the low-risk group demonstrated a lower Tumor Immune Dysfunction and Exclusion (TIDE) score and exhibited greater sensitivity to certain clinical therapeutic agents. Moreover, ARL6IP4 was found to be upregulated in CRC and underwent LLPS in live cells.

**Conclusion:**

We constructed and validated an eight-LRG risk signature, which can be employed to predict prognosis, characterize the immune landscape, and assess drug sensitivity in CRC.

## Introduction

Colorectal cancer (CRC), a prevalent malignancy within the digestive system, represents the second leading cause of cancer-related mortality globally (1, 2). Although significant advancements have been achieved in therapeutic strategies, the prognosis for CRC patients remains dismal (3, 4). Accurate prognostic assessment is of critical importance in clinical practice, as it guides the implementation of individualized treatment strategies. The tumor-node-metastasis (TNM) staging system is widely utilized for prognosis prediction in CRC, but it fails to account for the significant heterogeneity in clinical outcomes observed among patients within the same TNM stage (5, 6). In recent years, advancements such as next generation sequencing (NGS), liquid biopsy techniques including circulating tumor DNA (ctDNA)/circulating free DNA (cfDNA), circulating tumor cells (CTCs) as well as deep learning methodologies have begun to rectify some limitations inherent in traditional staging system (7–9). However, challenges related to standardization of testing procedures and/or cost-related constraints arise. Within the context of precision medicine, an exclusive reliance on either conventional pathological staging or novel molecular biomarkers significantly restricts the ability to predict clinical outcomes with accuracy. Consequently, developing an integrated prognostic signature that incorporates a multi-gene panel is imperative to enhance the accuracy of outcome prediction in patients with CRC.

Liquid-liquid phase separation (LLPS) is a phenomenon resulting from the weak interactions among biomolecules and leads to the formation of membraneless organelles, also known as biomolecular condensates (10, 11). It plays a critical role in multiple cellular processes, including genome stability, DNA damage and repair response, transcriptional regulation, and signal transduction (12–14). Dysregulation of LLPS can disrupt these processes, thereby contributing to cancer initiation and progression (15). Furthermore, emerging studies suggest that LLPS is linked to cancer patient prognosis, highlighting its potential as a prognostic biomarker and therapeutic target. Recent studies demonstrate that molecules like EphA2, MEX3A and MRNIP undergo LLPS and are highly expressed in CRC patients, with elevated levels associated with poor prognosis (16–18). A growing number of studies have begun to construct prognostic signatures based on LLPS-related genes (LRGs) across various types of cancer (19–21). However, the investigation of CRC remains insufficiently explored.

In this study, we used the The Cancer Genome Atlas (TCGA) database to develop a LRGs risk signature in the training cohort, and the prognostic significance of this signature was validated in the testing cohort and Gene Expression Omnibus (GEO) database. Moreover, the risk score as an independent prognostic factor was combined into a nomogram for assessing survival in CRC patients. Then, two groups were identified with distinct clinicopathological features, immune cell infiltration, immune function, therapeutic response, and mutational landscape. In particular, the expression and phase separation capability of ARL6IP4 were confirmed by experiments. Taken together, our study constructed a novel LRGs prognostic signature and shed new light on prediction of prognosis, immune landscape and drug sensitivity in CRC.

## Materials and methods

### Data and resources

RNA-sequencing data, corresponding clinicopathological characteristics, and mutation data of CRC were retrieved from the TCGA website (https://portal.gdc.cancer.gov/). GSE39582, was downloaded from the GEO website (https://www.ncbi.nlm.nih.gov/geo/). Patients lacking survival information or transcriptome profiling data were excluded. We obtained a total of 542 patients with CRC from TCGA for the purpose of signature construction and 557 patients from GSE39582 for external validation. The R package “sva” was used to adjustment for the batch effect between TCGA-CRC and GSE39582. The list of LRGs was selected from the DrLLPS (http://llps.biocuckoo.cn/), an online database that has incorporated 150 scaffolds, 987 regulators and 8148 potential clients (22). The 3611 LRGs identified in Homo sapiens were screened for subsequent studies.

### Differential expression analysis of LRGs and functional annotation

We used the “VennDiagram” package to screen for common genes between genes in the TCGA-CRC cohort and LRGs. The R package “limma” was applied to screen differentially expressed LRGs between the CRC and adjacent normal samples in the TCGA-CRC cohort. |log_2_FC|>1 and false discovery rate (FDR) <0.05 were considered as filtered criterion. The “pheatmap” package were utilized for generating differentially expressed LRGs heatmap. Principal component analysis (PCA) was performed to evaluate the distribution of samples. Gene Ontology (GO) and Kyoto Encyclopedia of Genes and Genomes (KEGG) were utilized by “clusterProfiler” package to explore the potential function and pathway in differentially expressed LRGs.

### Construction and verification of LRGs risk signature

By excluding genes that were not present in both GEO and TCGA, 773 differentially expressed LRGs were screened. A total of 542 CRC patients from TCGA were randomly divided into two independent groups in a 1:1 ratio using a computer-generated random within the R environment, creating a training cohort and a testing cohort. Then, we evaluated whether there were any statistically significant differences in clinical characteristics (including age, gender, tumor stage, T, N, and M) between the two cohorts. In the training cohort, univariate Cox regression was applied to choose differentially expressed LRGs having prognostic value. In the univariate Cox regression analysis, p<0.01 was deemed to be statistically significant. Next, we applied the least absolute shrinkage and selection operator (LASSO) Cox regression using the R package “glmnet” to eliminate overfitting and reduce the number of prognostic genes, with the penalty parameter tuned through 10-fold cross-validation. Finally, we performed multivariate Cox regression analysis to create a novel prognostic signature comprising eight genes. We calculated the risk score of each sample using the following formula: Risk score = $\sum_{i=1}^{n}\left(Coef\;{\text{gene}}_{\text{i}}\times \text{expression of }{\text{gene}}_{\text{i}}\right)$. Coefi and Expi stood for coefficient and expression associated with each signature gene, respectively. According to the median value of the risk score calculated from the training cohort, the entire patients were stratified into low-risk and high- risk groups. PCA was employed using the R package “scatterplot3d” and “limma” to assess whether the risk score could serve as a meaningful signature for classifying patients.

We used Kaplan-Meier (K-M) analysis and Receiver operating characteristic (ROC) analysis to confirm the predictive ability of this risk signature in the training, testing and entire cohort. Scatter plots, risk curves, and heatmaps were used to evaluate the differences between high-risk and low-risk groups in the training, testing and entire cohort. Additionally, the predictive performance of the signature was externally validated using GSE39582, applying the same calculation method and cut-off value to classify patients into high-risk and low-risk groups.

### Correlations between the LRGs signature and clinical factors

To evaluate the prognostic value of the risk score, univariate and multivariate Cox regression analyses were applied to screen for independent risk factors significantly related to survival (p<0.05). Using the “timeROC” package in R, ROC curve was applied to assess sensitivity and specificity of the risk score and clinical factors. Using the R packages “survival” and “rms”, a nomogram model was developed to predict the survival time of CRC patients based on the risk score and various clinical features (Gender, risk, age, and tumor stage). The nomogram assigned a score to each variable, and the total score for each patient was used to estimate survival time. Moreover, we plotted calibration curves to evaluate the consistency between actual and predicted probabilities for the 1-, 3-, and 5-year overall survival (OS). The concordance index (C-index) was utilized to evaluate the predictive ability of the nomogram.

### Immune characteristic analysis and tumor mutation burden analysis

The differences in 22 immune cell types between the high-risk and low-risk groups were evaluated using the CIBERSORT algorithm. The Wilcoxon rank-sum test assessed the risk score differences between the two groups. Furthermore, the relationship between the risk score and immune infiltrating cells was assessed using Spearman correlation analysis. The “estimate” R package was used to calculate stromal, immune, and ESTIMATE scores between two risk groups. We used R package “maftools” to draw a waterfall which could indicate mutation landscape in our samples. The “survival” package was used to observe the relationship among TMB, risk score, and patient survival.

### Immunotherapy response and drug sensitivity

The Tumor Immune Dysfunction and Exclusion (TIDE) algorithm (http://tide.dfci.harvard.edu/) was employed to assess the responsiveness of two groups to immunotherapy. The “OncoPredict” package in R was used to analyze the differences in drug sensitivity between the two groups (p<0.001). The detailed data were obtained from Genomics of Drug Sensitivity in Cancer (GDSC) database (https://www.cancerrxgene.org/).

### Clinical specimens, RNA extraction and qRT–PCR

A cohort comprising ten pairs of CRC tissues and corresponding uninvolved tissues was collected from CRC patients who underwent radical resection or palliative resection at the Department of Gastrointestinal Surgery of the Affiliated Hospital of Xuzhou Medical University, and consent was obtained from all patients. Total RNA was extracted from the cohort using the RNA Isolater Total RNA Extraction Reagent (Vazyme, Nanjing, China). A 1000ng aliquot of RNA was subjected to reverse transcription using the SweScript RT I First Strand cDNA synthesis kit (Servicebio, Wuhan, China). The cDNA analysis was performed via quantitative real–time polymerase chain reaction (qRT–PCR) with the ChamQ SYBR qPCR Master Mix (Vazyme, Nanjing, China) on a LightCycler 96 system (Roche, Switzerland). GAPDH was used as an endogenous control. The primers used are as follows: ARL6IP4 Forward: TGACCAAGGAGGAGTGGGAT, ARL6IP4 Reverse: CCTCTAGGACCTCGCCATCT; GAPDH Forward: CGGAGTCAACGGATTTGGTCGTAT and Reverse: AGCCTTCTCCATGGTGGTGAAGAC. The relative RNA expression levels were quantified using the 2^-ΔΔCT^ method, with GAPDH utilized as the reference gene for RNA normalization.

### Phase separation assay in CRC cells

ARL6IP4 sequences were introduced into the pcDNA3.1-EGFP-C2 vector (YouBio, VT2050). HCT116 and LoVo cells were obtained from the Cell Bank of the Chinese Academy of Sciences (Shanghai, China), and cultured in glass-bottom dishes and subsequently transfected with ARL6IP4-EGFP plasmids. The EGFP-tagged puncta and Z-Stack images of transfected cells were acquired using a Leica STELLARIS 5 confocal microscope (CLSM, Leica STELLARIS 5, Germany) equipped with a 63× oil immersion objective, then reconstructed in three dimensions using the 3D reconstruction module of LAS X software. Fluorescence Recovery After Photobleaching (FRAP) was conducted on the region of interest (ROI) using a 488 nm laser at 100% power for 10 seconds to achieve complete or partial fluorescence loss. Time-lapse images were acquired every second over a 1-minute period following photobleaching. Fluorescence intensities within the ROI were normalized to their respective pre-bleach values, and data were exported for analysis. Recovery curves were plotted from normalized fluorescence data of three independent replicates using GraphPad Prism 9.

### Statistical analysis

Statistical analyses and visualization were carried out using R software (version 4.3.2). The independent prognostic factor for OS was assessed by univariate and multivariate Cox regression analyses. The Student’s t-test or the Wilcoxon test was used to compare the data from different groups. The Chi-square test was utilized to investigate the relationship between risk groups and clinicopathological characteristics. The log-rank test was used for K-M analysis to evaluate survival curves of specific groups. The significance level of correlation was explored via the Spearman method.

## Results

### Differential expression analysis of LRGs and functional annotation

**Figure 1:**
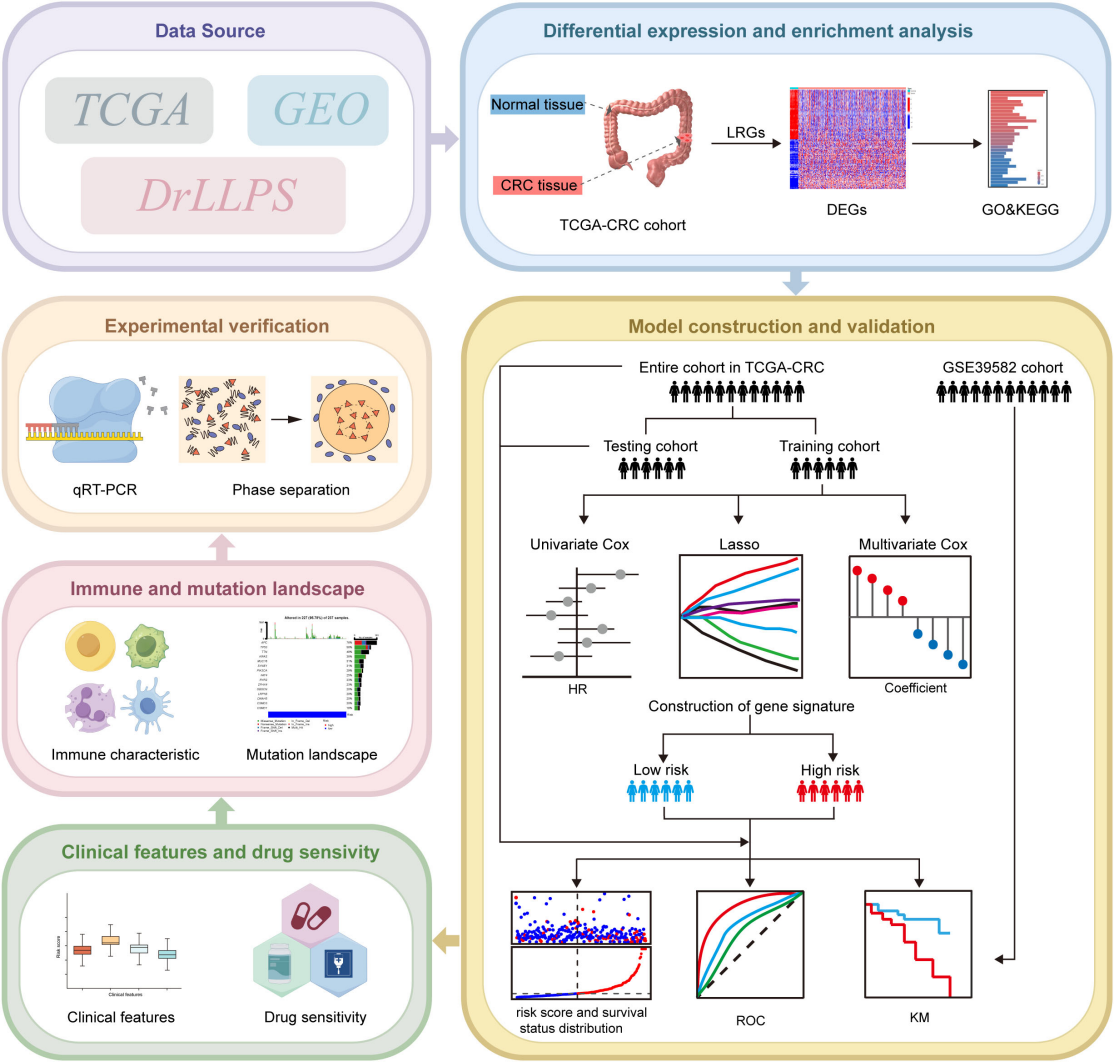
Schematic representation of the study design. Certain images were created by Figdraw.

**Figure 2:**
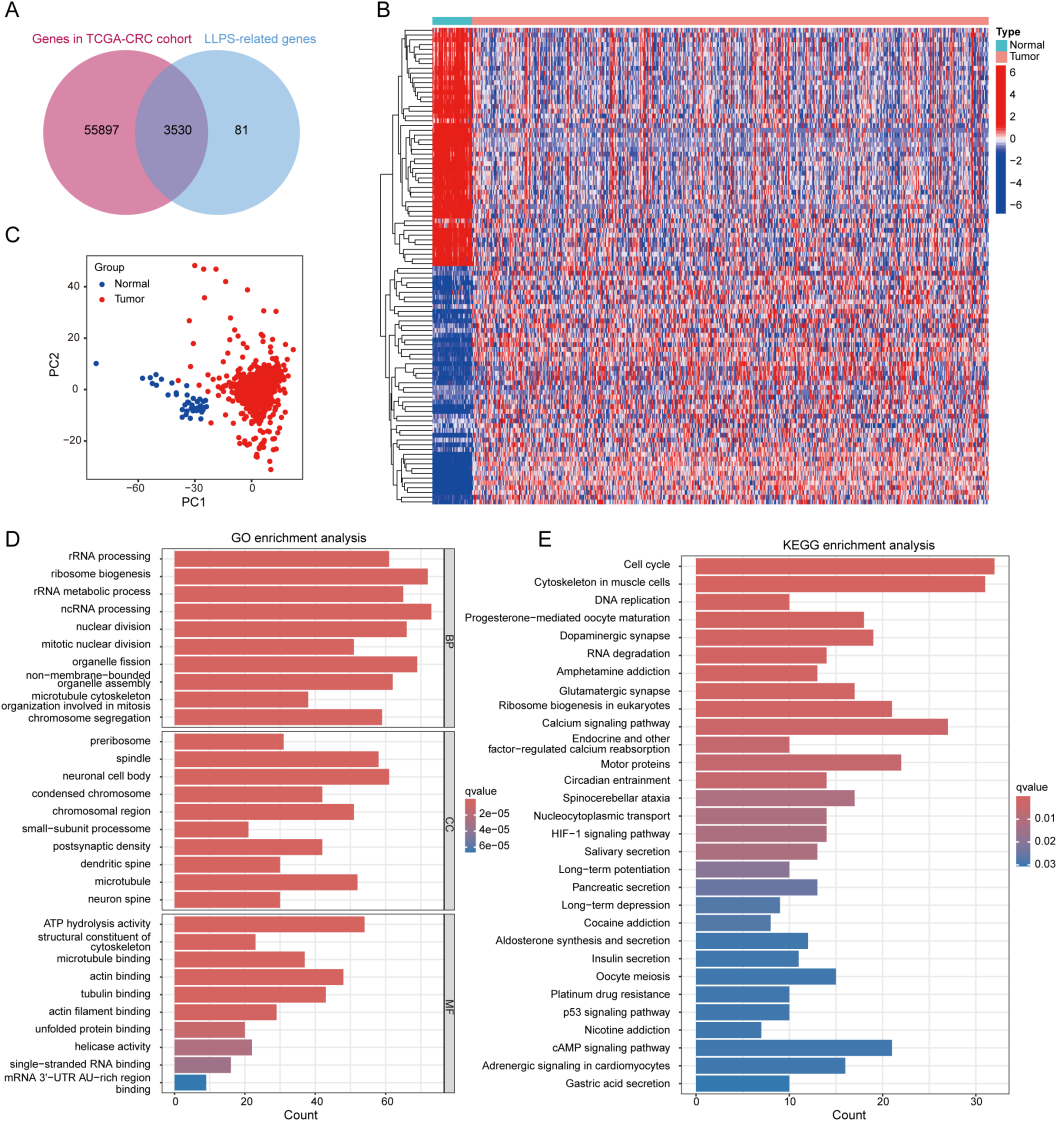
Differential expression analysis of LRGs and functional annotation. **(A)** The Venn diagram illustrating the intersection of genes identified in the DrLLPS database and TCGA-CRC. **(B)** Heatmap illustrating the varying expressions of LRGs in normal and tumor based on the following criteria: adjusted P-value<0.05 and |log_2_FC|>1. **(C)** PCA conducted on the differentially expressed LRGs. **(D)** GO enrichment analysis was conducted on differentially expressed LRGs with respect to biological processes (BP), cellular components (CC), and molecular functions (MF). **(E)** KEGG enrichment analysis of the differentially expressed LRGs.

The flow chart of our study is shown in **Figure 1**. By taking the intersection of genes in TCGA-CRC cohort and LRGs in DrLLPS database, 3530 LRGs were obtained (**Figure 2A**). Then, a total of 3526 genes which had available gene expression data in TCGA-CRC cohort were screened out for subsequent differential expression analysis between CRC samples and normal samples. According to the threshold of FDR<0.05 and |log_2_FC|>1, we distinguished 828 differentially expressed LRGs, among which 299 were downregulated and 529 were upregulated. The heatmap illustrated the top 50 upregulated and downregulated differentially expressed LRGs, respectively (**Figure 2B**). PCA was used to show that there was an obvious difference between the normal and CRC samples based on these differentially expressed LRGs (**Figure 2C**). GO and KEGG pathway annotation analyses were carried out to uncover potential biological functions of the differentially expressed LRGs in CRC. GO analysis showed that these differentially expressed LRGs were associated with the process of LLPS, such as the non-membrane-bounded organelle assembly (**Figure 2D**). KEGG analysis showed that tumor-related pathways were enriched by these differentially expressed LRGs, such as cell cycle, DNA replication, HIF-1 signaling pathway, platinum drug resistance, and p53 signaling pathway (**Figure 2E**).

### Construction and verification of a prognostic risk signature based on LRGs

**Figure 3:**
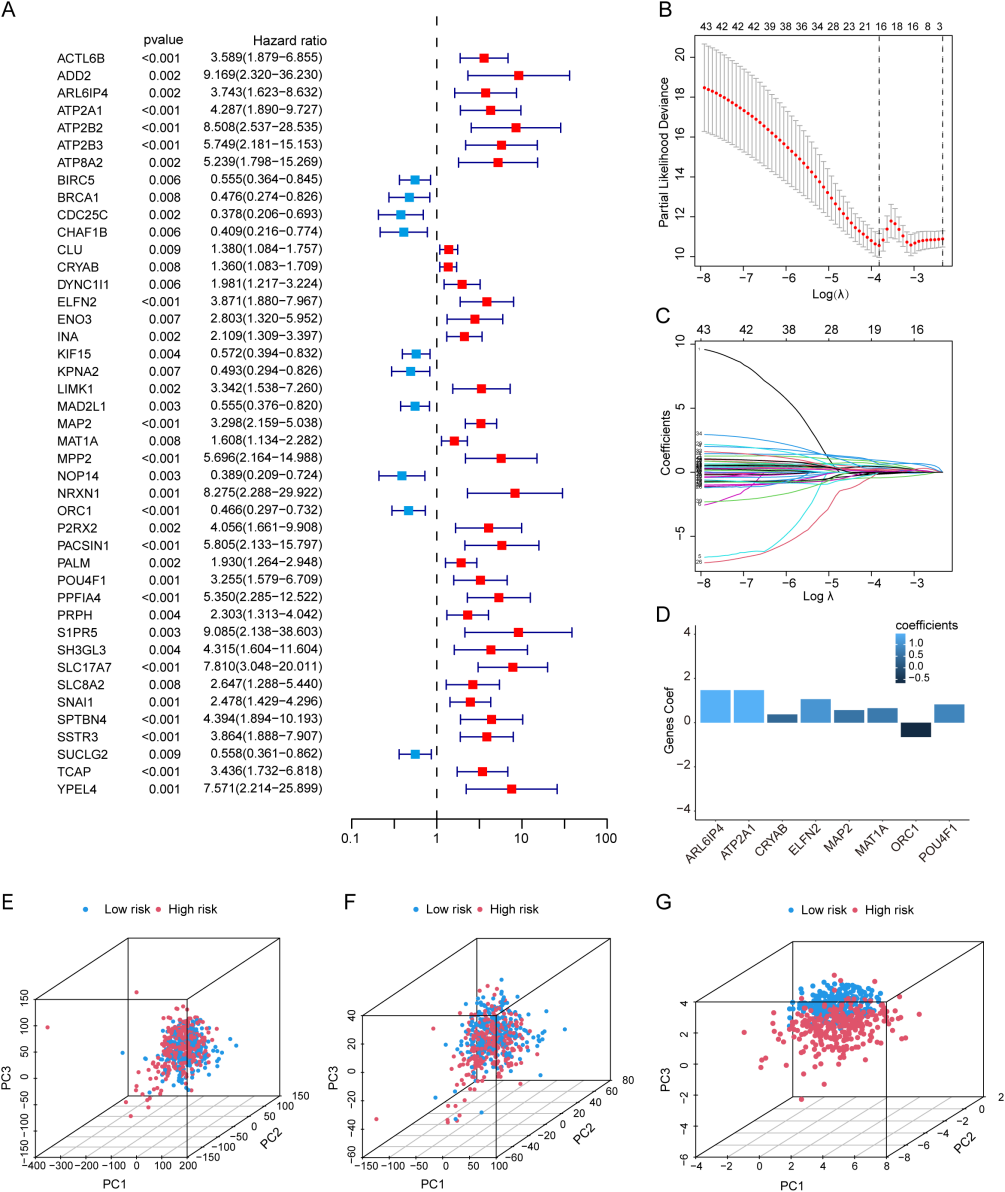
Construction of a LLPS-related prognostic risk signature. **(A)** Univariate Cox regression analysis of LRGs in TCGA-CRC. **(B, C)** LASSO Cox regression analysis for the development of a risk signature. **(D)** Multivariate Cox regression analysis of prognostic genes identified through LASSO regression. **(E–G)** PCA presenting the separation of low-risk and high-risk groups across all genes **(E)**, LRGs **(F)**, and risk genes **(G)**.

**Figure 4:**
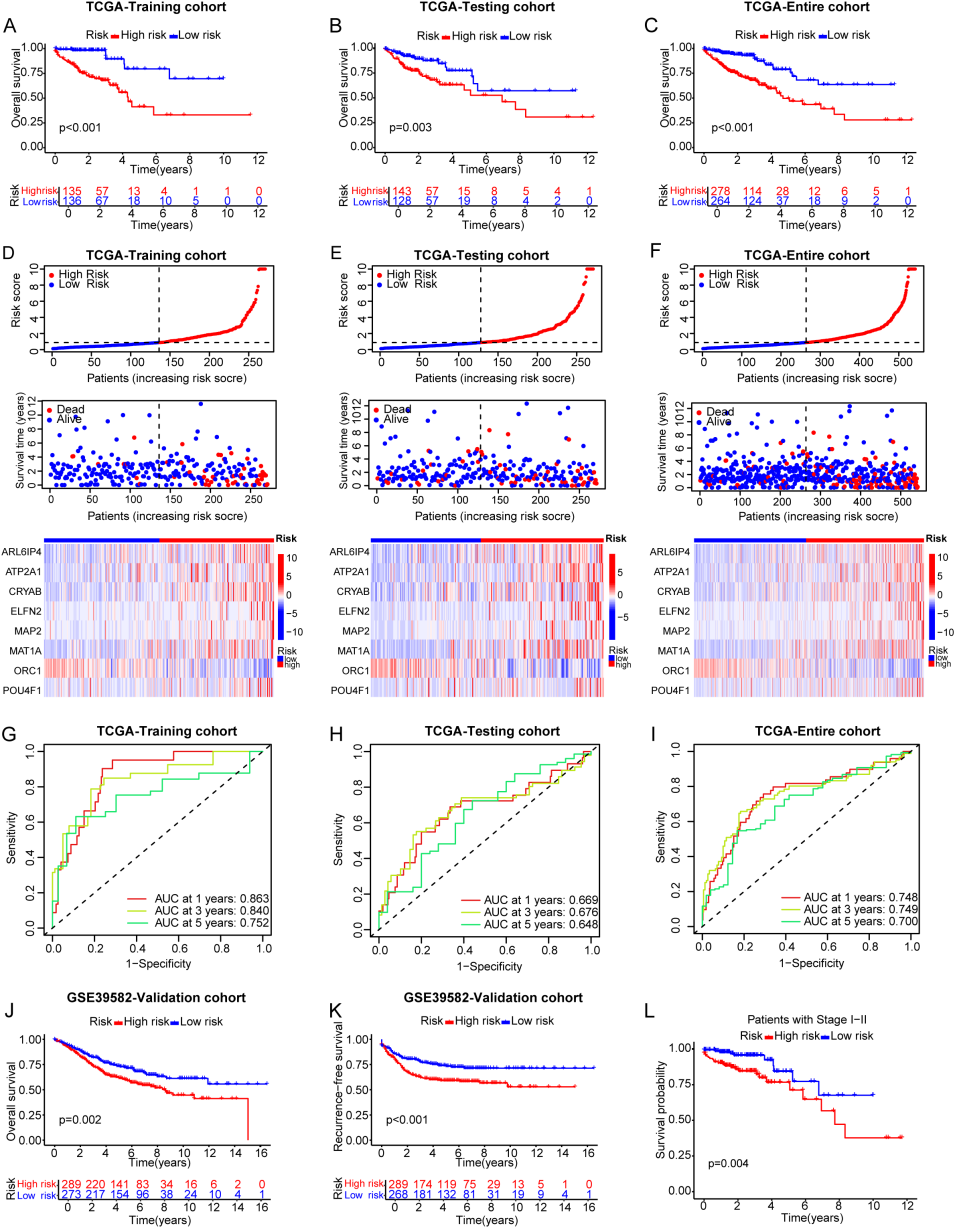
Prognostic performance and verification of the LRGs risk signature. **(A–C)** K-M curves illustrating OS for the two risk groups within the TCGA-CRC across the training **(A)**, testing **(B)**, and entire **(C)** cohorts. **(D–F)** The ranked dot and scatter plots illustrate the distribution of risk scores alongside patient survival outcomes, while the heatmap depicts the characteristics of eight genes across the training **(D)**, testing **(E)**, and entire **(F)** cohorts. **(G–I)** ROC curves were utilized to assess the sensitivity and specificity of 1-, 3-, and 5-year survival rates based on the risk score in the training **(G)**, testing **(H)**, and entire **(I)** cohorts. **(J)** The K-M curves illustrating OS for the two risk groups within the GSE39582. **(K)** The K-M curves illustrating RFS for the two risk groups within the GSE39582. **(L)** The K-M curve illustrated the prognosis for the tumor stage I-II subgroup.

The CRC samples from the TCGA database were randomly divided into a training cohort (50%, n=271 cases) and a testing cohort (50%, n=271 cases). Additionally, the clinical characteristics between the two cohorts were compared and showed no difference (**Supplementary Table S1**). Next, to construct a novel prognostic signature, a univariate Cox regression analysis was carried out in the training cohort to preliminarily filter for genes associated with prognosis. 43 genes were identified as prognostic genes in CRC, among which ten genes were deemed as protective genes with HR<1, and the rest 33 genes were deemed as risk genes with HR>1 (**Figure 3A**). Furthermore, LASSO Cox regression was performed to reduce overfitting (**Figures 3B, C**), and followed by multivariate Cox regression analysis (**Figure 3D**). We obtained eight genes ARF Like GTPase 6 Interacting Protein 4 (ARL6IP4), ATPase sarcoplasmic/endoplasmic reticulum Ca2+ transporting 1 (ATP2A1), crystallin alpha B (CRYAB), extracellular leucine rich repeat and fibronectin type III domain containing 2 (ELFN2), microtubule associated protein 2 (MAP2), methionine adenosyltransferase 1A (MAT1A), origin recognition complex subunit 1 (ORC1), and POU class 4 homeobox 1 (POU4F1) which have influence on the OS of CRC patients to build a prognostic signature. Then, the risk score for each patient with CRC was computed based on the following algorithm: Risk score = $\sum_{i=1}^{n}\left(Coef\;{\text{gene}}_{\text{i}}\times \text{expression of }{\text{gene}}_{\text{i}}\right)$. Consequently, the risk score for each sample was ascertained through the application of the aforementioned formula. In the light of the median risk score in the training cohort, patients were categorized into the high-risk and low-risk groups. We explored differential expression analysis of eight prognostic signature genes in the high-risk and low-risk groups (**Supplementary Figure S1A–H**). PCA was used to illustrate the spread of patients across all genes in TCGA-CRC (**Figure 3E**), LRGs (**Figure 3F**), and risk genes (**Figure 3G**). Notably, CRC patients were distributed in distinctly different directions based on risk genes, which suggested that the risk signature could likely distinguish between high-risk and low-risk groups. K-M analysis revealed that both OS (**Figure 4A**) and progression free survival (PFS) (**Supplementary Figure S2A**) in the high-risk group were significantly poor than that of the low-risk group. Risk score and survival status distributions demonstrated that the mortality rate rose in tandem with the elevation of the risk score in the training cohort, and the expression of the eight genes in high-risk and low-risk groups was exhibited in a heatmap (**Figure 4D**). Then, utilizing the same equation, we calculated the risk score for each patient in the testing cohort as well as the entire cohort, with these cohorts serving to validate the signature. The findings mirrored those observed within the training cohort (**Figure 4B, C, E, F**, **Supplementary Figures S2B, C**). Furthermore, ROC curves were used to assess the predictive power of the signature. In the training cohort, the area under the curves (AUCs) at 1, 3, and 5 years were 0.863, 0.840, and 0.752, respectively (**Figure 4G**). For the testing cohort, the AUCs at 1, 3, and 5 years were 0.669, 0.676 and 0.648 (**Figure 4H**), while in the entire cohort, they were 0.748, 0.749 and 0.700 (**Figure 4I**). These results highlighted the superior predictive accuracy of our signature. In addition, we introduced a GEO dataset (GSE39582), which served as an external validation cohort. The K-M curves of GSE39582 indicated that the prognosis for the high-risk group was poorer than that of the low-risk group, which aligns with the prognostic outcomes observed in the TCGA cohort (**Figures 4J–K**). Additionally, to assess the efficacy of the LRGs risk signature in predicting prognosis in patients with CRC, we stratified the entire patients into different subgroups according to clinical characteristics, including tumor stage I–II or tumor stage III–IV, male or female, younger(≤ 65 years) or older (>65 years), N0 or N1–N2, and M0 or M1(**Figure 4L**, **Supplementary Figures S2D–L**), These results affirmed the effectiveness and robustness of the risk signature in forecasting the survival outcomes of CRC patients.

### Independent prognostic significance and clinical features of the risk signature

**Figure 5:**
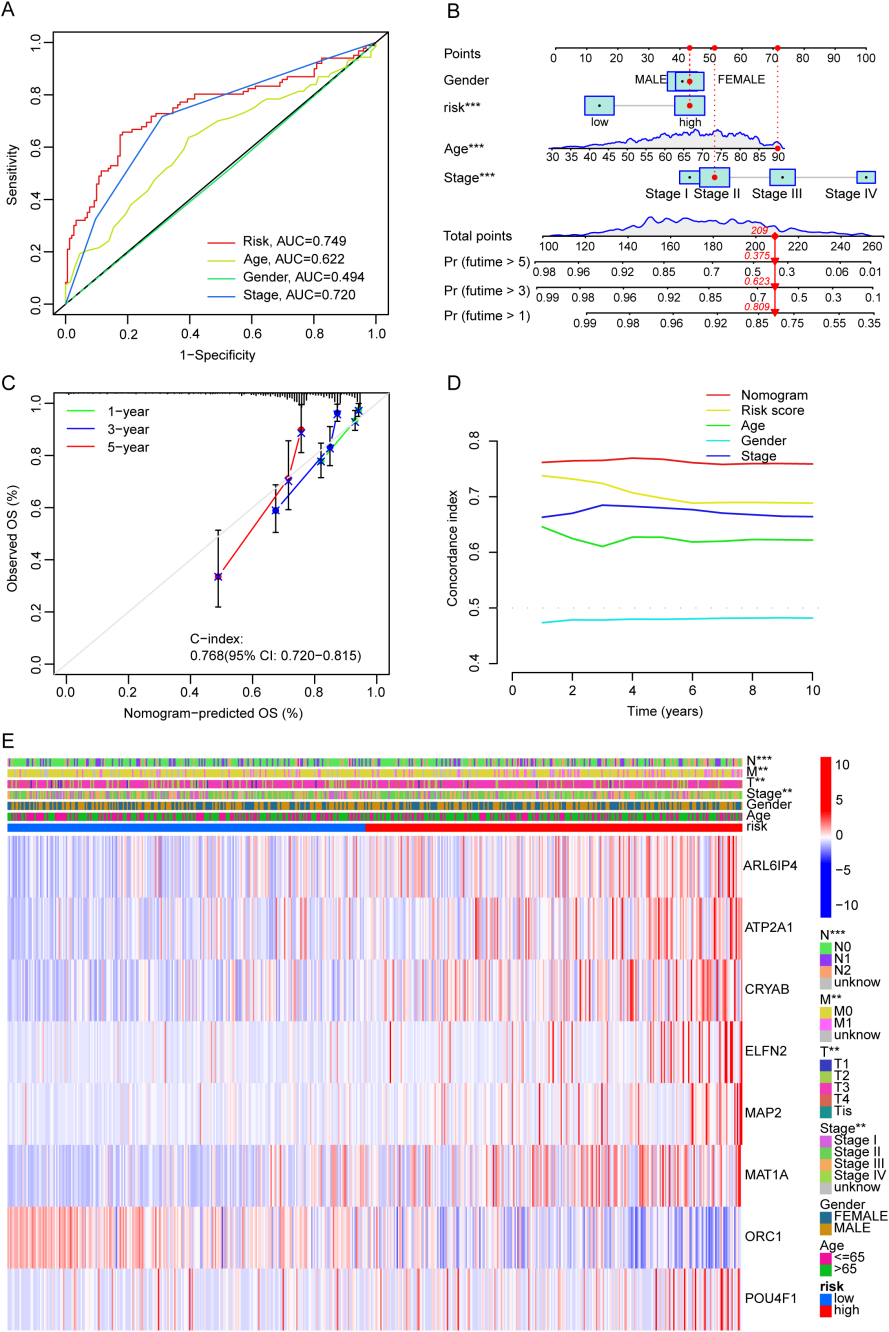
ROC curve analysis, Nomogram construction and clinical features of the risk signature. **(A)** Prognostic ROC curves for the risk score and clinical factors. **(B)** Development of a nomogram for predicting 1-, 3-, and 5-year survival rates in patients with CRC. **(C)** Calibration curve for assessing the accuracy of the nomogram. **(D)** C-index curve used to evaluate the performance of the nomogram predicting prognosis. **(E)** The heatmap illustrates the relationship between the expression levels of the eight genes and various clinicopathological characteristics. **p < 0.01, ***p < 0.001.

Univariate Cox regression analysis indicated that risk score, Age, tumor stage, and T, N and M stage were the significant prognostic factors for OS in CRC patients (**Supplementary Figure S3A**). Furthermore, multivariate analysis indicated that the risk score functioned as an independent prognostic marker for OS (HR 1.030, 95% CI 1.019-1.041). (**Supplementary Figure S3B**). Besides, ROC analysis showed that the AUC for risk score was 0.749, which was superior performance in predictive markers compared to tumor stage (AUC = 0.720) (**Figure 5A**). We created a prognostic nomogram with the gender, risk score, age, and tumor stage (**Figure 5B**). The calibration curves demonstrated that the nomogram’s predictions for 1-, 3-, and 5-year OS probabilities closely matched the ideal predictions (**Figure 5C**), and the nomogram model’s C-index was 0.768 (95% CI 0.720-0.815). The C-index value of the nomogram was significantly higher than other clinical characteristics (**Figure 5D**). These results implied that the nomogram demonstrated effective in forecasting patient prognoses and could be used as a tool for clinical decision-making. Additionally, the risk score was different in groups classified by tumor stage, T stage, N stage, and M stage (**Supplementary Figures S3C–H**). Heatmap showed the correlation between expression levels of the eight LRGs and clinicopathological features of CRC patients in the TCGA database (**Figure 5E**).

### Correlation between the risk signature and immune cell infiltration

**Figure 6:**
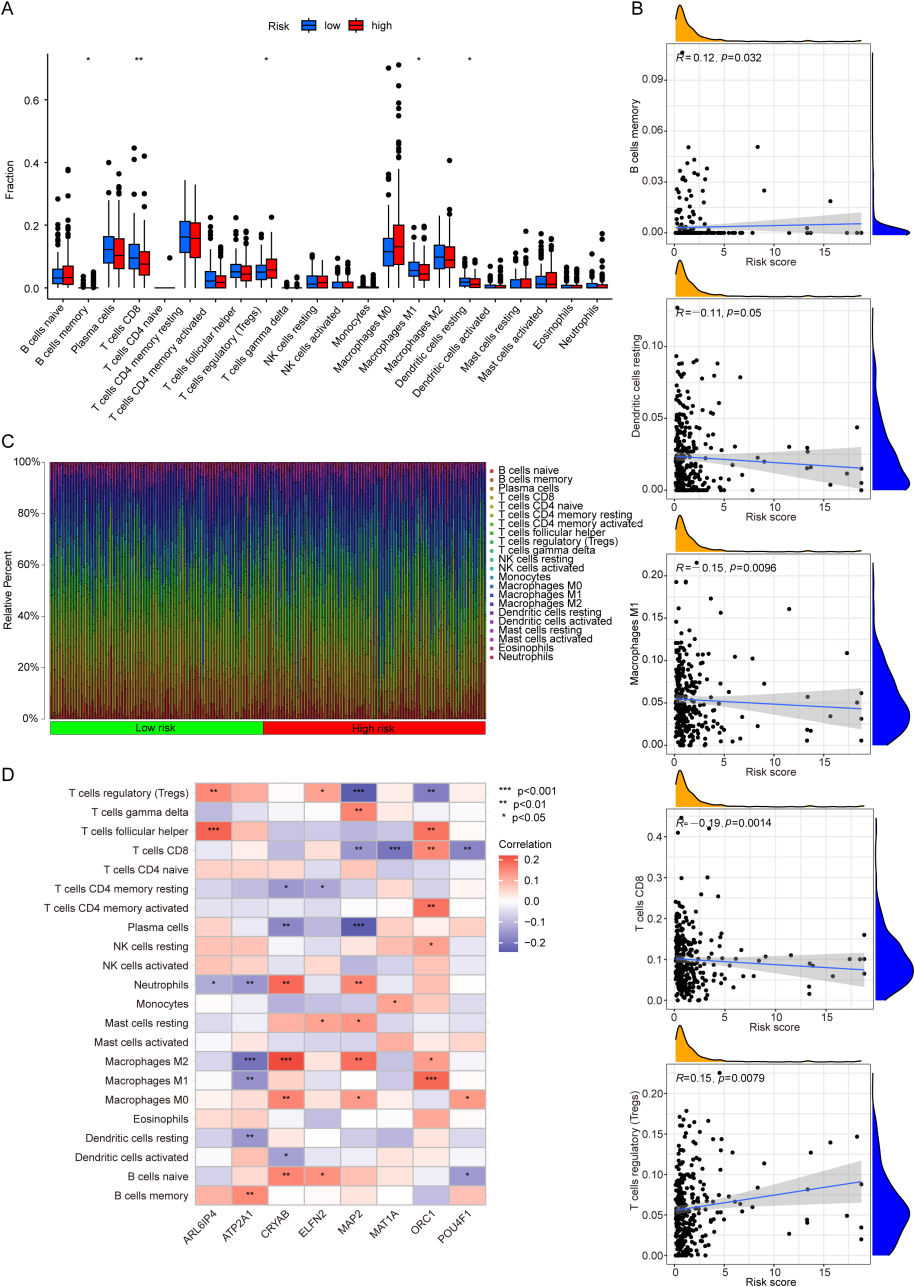
Correlation between the risk signature and immune cell infiltration. **(A)** Differential analysis of immune cell infiltration between the two groups. **(B)** Correlation analyses between infiltrating immune cell types and the risk scores. **(C)** Bar graphs illustrating the distribution of infiltrating immune cells between the high-risk and low-risk groups. **(D)** Correlation analyses between infiltrating immune cell types and the eight LRGs. *p < 0.05, **p < 0.01, ***p < 0.001.

The tumor immune microenvironment is widely acknowledged for its pivotal role in regulating cancer progression, clinical outcomes, and responses to treatment (23, 24). To explore the correlation between characteristics of immune cells and the risk signature, we first assessed the relative fractions of 22 tumor-infiltrating immune cells in each sample of CRC by the CIBERSORT algorithm. We observed more infiltration of B cells memory and T cells regulatory (Tregs), but less proportions of T cells CD8, Macrophages M1 and Dendritic cells resting in the high-risk group compared to the low-risk group (**Figure 6A**). These findings suggest that immune cell composition is an important factor contributing to poor prognosis in the high-risk group. Spearman correlation analyses demonstrated that the risk score was negatively associated with Dendritic cells resting, Macrophages M1and T cells CD8, but positively associated with B cells memory and Tregs (**Figure 6B**). The immune cells composition of each sample was shown in **Figure 6C**. In addition, **Figure 6D** illustrated that the eight genes were all associated with immune cells, suggesting that these genes may serve as potential targets for CRC immunotherapy.

### Association of the risk signature with immune characteristics and drug sensitivity

**Figure 7:**
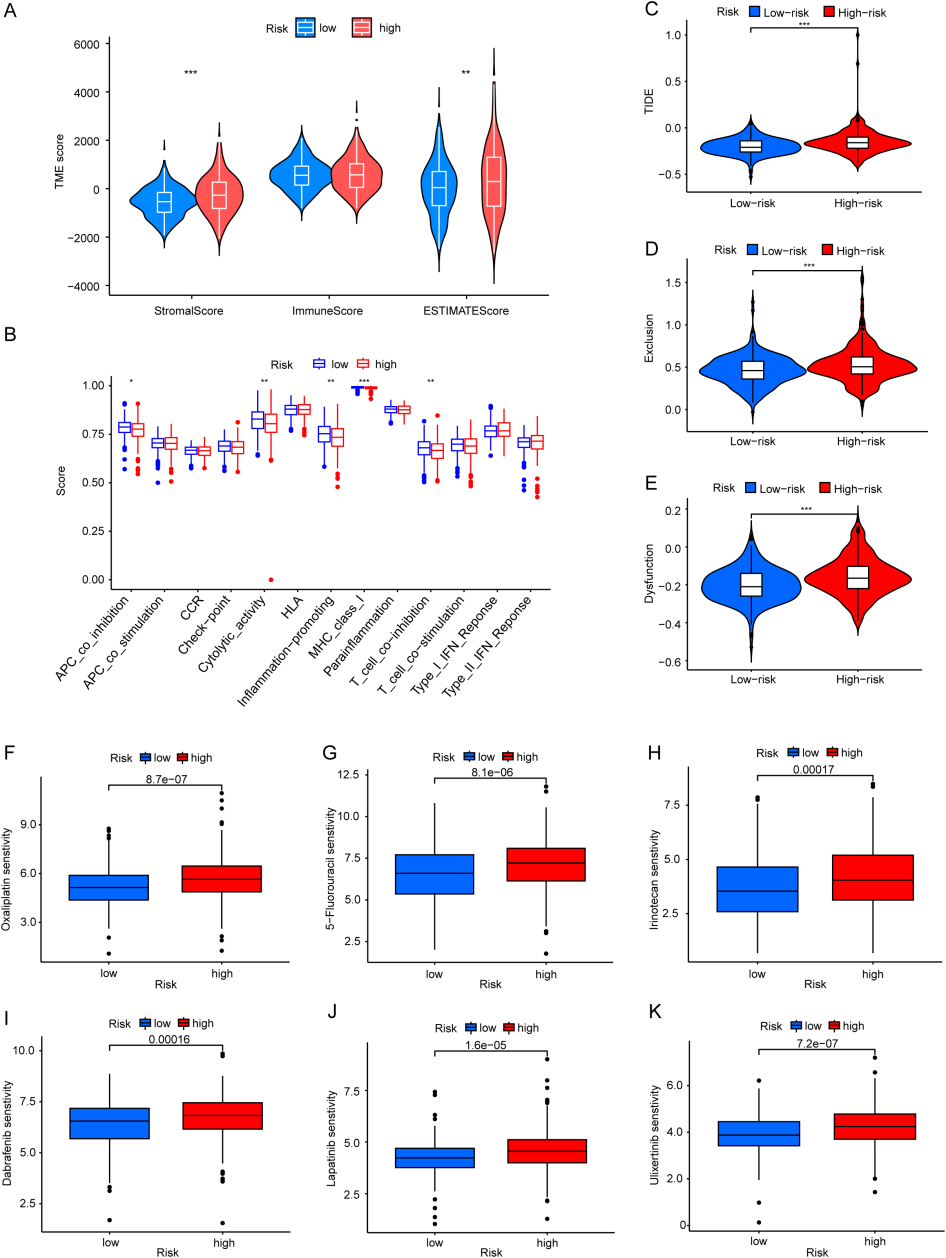
Immune characteristic and drug sensitivity analysis. **(A)** Differential analyses of stroma, immune, and ESTIMATE scores between the two groups. **(B)** Differential analyses of immune-related functions between two groups. **(C–E)** Comparison of TIDE **(C)**, Exclusion **(D)**, and Dysfunction **(E)** scores between the two groups. **(F–K)** The differences in drug sensitivity to Oxaliplatin **(F)**, 5-Fluorouracil **(G)**, Irinotecan **(H)**, Dabrafenib **(I)**, Lapatinib **(J)**, and Ulixertinib **(K)** between the two groups. *p < 0.05, **p < 0.01, ***p < 0.001.

ESTIMATE algorithm revealed that the high-risk group possessed elevated stromal score and ESTIMATE score than low-risk group, while no notable disparity was observed between the two groups regarding immune score (**Figure 7A**), suggesting that the infiltration of stromal cells markedly escalated as the risk score increased. The high-risk group showed a higher stromal score and poor prognostic outcomes, aligning with previous research findings (25, 26). Comparative analysis of immune related functions showed that the scores of APC co-inhibition, cytolytic activity, inflammation promoting, MHC class I and T cell co-inhibition were lower in the high-risk group (**Figure 7B**). Besides, a higher TIDE score was observed in the high-risk group compared to the low-risk group (**Figures 7C–E**). This indicated that CRC patients in high-risk group were more prone to immune evasion. Furthermore, we investigated the differences in drug sensitivity between the two risk groups. In terms of chemotherapy agents for CRC, oxaliplatin, 5-fluorouracil(5-FU), and irinotecan demonstrated greater efficacy in the low-risk group compared to the high-risk group (**Figures 7F–H**). Regarding targeted therapies for CRC, the low-risk group exhibited heightened sensitivity to dabrafenib, lapatinib, and ulixertinib relative to the high-risk group (**Figures 7I–K**). Additionally, there were differential sensitivities observed for another 101 drugs between the two risk groups (**Supplementary Figure S4, 5**).

### Correlation between the risk signature and TMB

**Figure 8:**
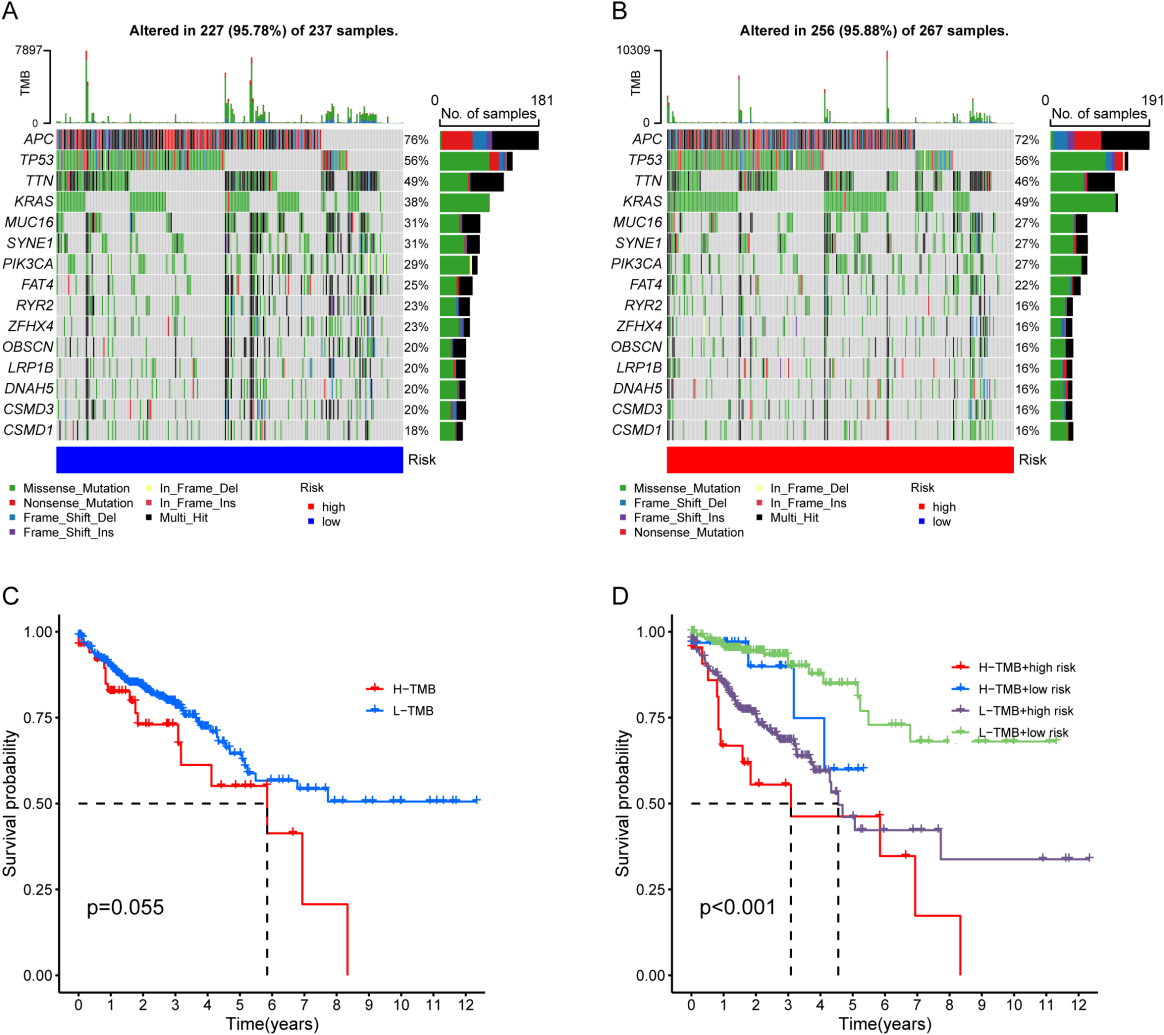
Correlation between the risk signature and TMB. **(A, B)** The top 15 mutated genes were presented in the low-risk and high-risk groups, respectively. **(C)** K-M analysis between the high-TMB and low-TMB groups. **(D)** K-M curves show prognosis combining risk score and TMB.

We analyzed the mutation landscape of CRC in both high-risk and low-risk groups. Maftools analysis results showed that the top 15 high-frequency mutated genes in low-risk and high-risk groups, respectively (**Figures 8A, B**). The most common mutated gene was APC. Previous studies have revealed that high TMB predicted poor prognosis across various types of cancers. In our assessment of TMB’s prognostic value, we observed that the survival prospect of patients in the high-TMB group and low-TMB group was no significant, but high-TMB group tended to have worse outcome (**Figure 8C**). Next, CRC patients were categorized into four distinct groups for a survival analysis that considered both TMB and risk score, including low-TMB + low-risk, low-TMB + high-risk, high-TMB+ low-risk, and high-TMB + high-risk. The finding showed that patients in the low-TMB + low-risk group had a more favorable survival outcome compared to patients in the high-TMB + low-risk group (**Figure 8D**).

### Expression level and phase separation capability of ARL6IP4 in CRC

**Figure 9:**
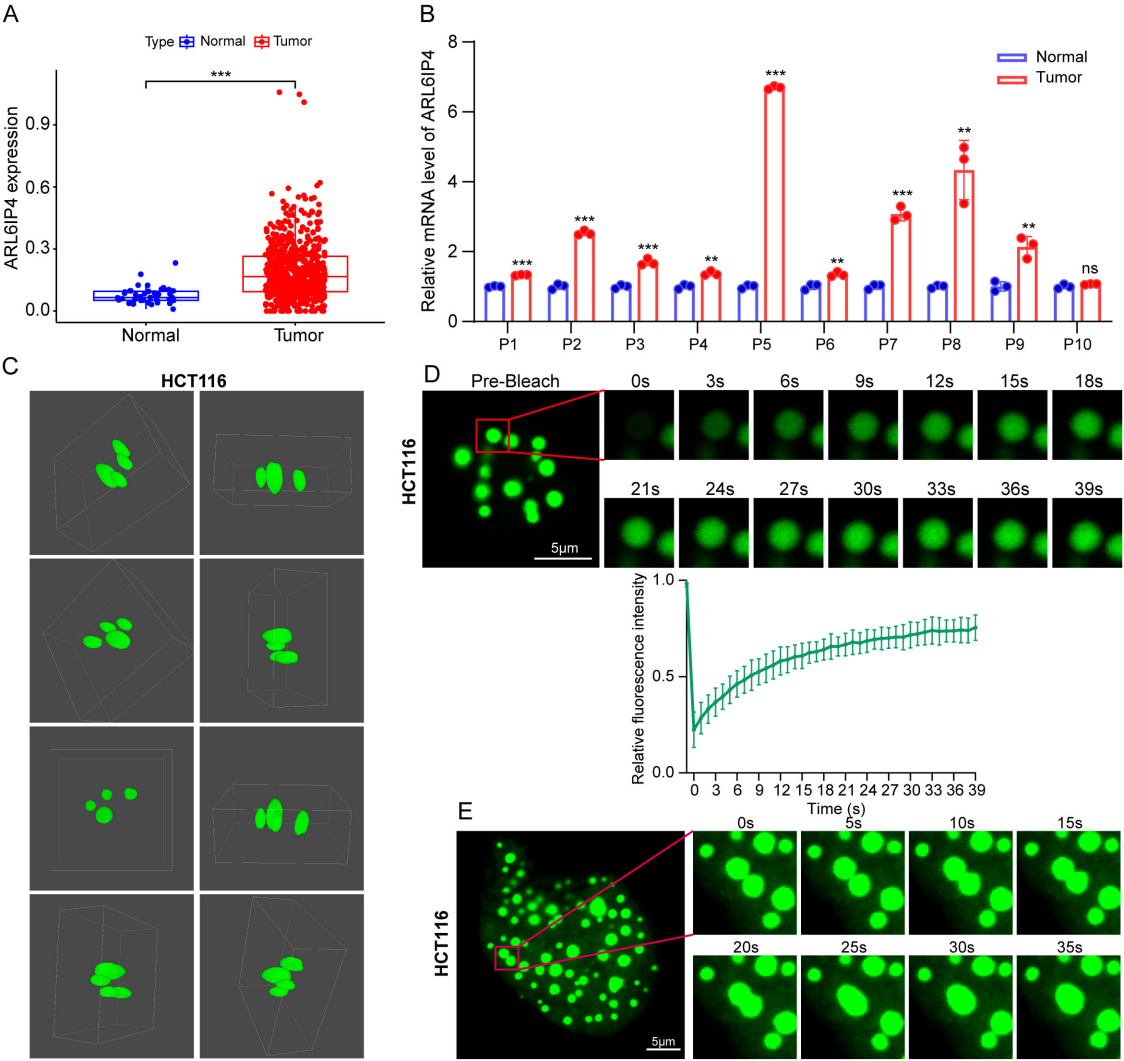
Expression level and phase separation of ARL6IP4 in CRC. **(A)** The mRNA level of ARL6IP4 in CRC and normal tissues from TCGA database. **(B)** The mRNA level of ARL6IP4 in ten pairs of CRC tissues and uninvolved tissues. **(C)** 3D-reconstructed images of live HCT116 cells transfected with EGFP-ARL6IP4 plasmid were acquired using a confocal laser scanning microscope. **(D)** Images (top) and quantitative analysis (bottom) of EGFP-ARL6IP4 FRAP were presented in HCT116 cells. Data was expressed as Mean ± SE, with n = 3 independent biological replicates. **(E)** Two EGFP-ARL6IP4 droplets in HCT116 cells underwent fusion to create a larger droplet. **p < 0.01, ***p < 0.001; ns, not significant.

To investigate whether the gene utilized in constructing the risk signature for this study underwent phase segregation, we selected ARL6IP4, which had the highest coefficient in the multivariate Cox regression analysis, for experimental verification. Firstly, the upregulation of ARL6IP4 was confirmed in the TCGA-CRC cohort (**Figure 9A**). Furthermore, we assessed the expression levels of ARL6IP4 in ten paired fresh frozen tissue specimens using qRT–PCR. The results indicated that ARL6IP4 was significantly upregulated in majority of CRC tissues when compared to uninvolved tissues (**Figure 9B**). Subsequently, following the ectopic transfection of ARL6IP4-EGFP plasmid, we observed that ARL6IP4 forms droplet-like structures within the live CRC cells using 3D confocal microscopy (**Figure 9C**, **Supplementary Figure S6A**). To assess the fluidity of ARL6IP4 condensates, we performed FRAP experiments. The results demonstrated that the fluorescence within ARL6IP4 condensates recovers rapidly after photobleaching (**Figure 9D**, **Supplementary Figure S6B**). Furthermore, through time-lapse imaging, we observed that the punctate structures exhibited fusion behavior in live CRC cells (**Figure 9E**, **Supplementary Figure S6C**). In summary, our findings demonstrated that ARL6IP4 was upregulated in CRC and underwent phase separation.

## Discussion

Although existing prognostic methods for CRC play a crucial role in clinical practice, their limitations impede the progress of precision medicine. The TNM staging system, despite its widespread application, demonstrates suboptimal prognostic value due to significant tumor heterogeneity and complex biological factors. Notably, survival outcomes can vary considerably even among patients within the same stage; for instance, some stage IIIa patients may exhibit better prognoses than certain subgroups classified as stage II (27). Beyond TNM staging, molecular markers such as MSI status and KRAS/BRAF mutations have emerged as critical prognostic factors. Patients with pMMR/MSI-L/MSS tumors typically experience poorer outcomes compared to those with dMMR/MSI-H CRC (28). However, despite their significance, MSI/MMR testing remains underutilized, even among high-risk populations (29). Similarly, KRAS and BRAF mutations are linked to poorer PFS and OS in metastatic CRC (30). However, the utility of these factors as independent prognostic indicators is constrained by their applicability to patients with specific mutations and their limitations in providing personalized risk stratification. The consensus molecular subtype (CMS) classification offers a more nuanced understanding of CRC heterogeneity. For example, CMS4 is characterized by significant stromal infiltration and activation of angiogenesis, which correlates with unfavorable clinical outcomes (31). However, integrating CMS into routine clinical practice faces barriers, such as the complexity and cost of high-throughput genomic technologies (32). Additionally, the advent of liquid biopsy has established CTCs as a reliable independent predictor of adverse clinical outcomes in CRC (33). Nonetheless, the clinical utility of CTCs is limited by inadequate detection sensitivity (34). Collectively, these limitations highlight the pressing need for innovative biomarkers and integrated prognostic signatures that amalgamate clinical and molecular data. Such signatures would not only improve the accuracy of outcome predictions but also facilitate the development of personalized therapeutic strategies for patients with CRC.

Therefore, we developed a risk signature comprising eight LRGs to predict the outcomes of patients with CRC. In this study, regardless of whether in the training cohort, testing cohort, or the entire TCGA cohort, as well as in the external GEO database cohort, patients classified into the high-risk group exhibited significantly shorter survival times compared to those in the low-risk group. Additionally, the ROC analysis demonstrated that the AUC for the risk score outperformed TNM stage as a predictive marker, which is a critical indicator in current prognostic assessments. Furthermore, we conducted both univariate and multivariate analyses, demonstrating that our risk signature served as an independent prognostic factor. Subsequently, we constructed a nomogram by integrating the risk score with clinical characteristics. This approach aimed to enhance predictive accuracy and facilitate its application in clinical practice. Therefore, our signature, which is based on the cost-effective detection of only eight genes, not only provides a more personalized prognostic assessment but also offers additional molecular stratification information to address the prognostic heterogeneity among patients within the same TNM stage. Moreover, given that gene expression changes may precede anatomical alterations (eg tumor size and lymph node metastasis), our signature offers a significant advantage in prognostic prediction for early-stage patients with CRC. Furthermore, our risk signature enables precise therapeutic stratification for all patients with CRC, not limited to those with gene mutations.

In our eight signature genes (ARL6IP4, ATP2A1, CRYAB, ELFN2, MAP2, MAT1A, ORC1, and POU4F1), prior research has elucidated the expression and function of several genes involved in the initiation and progression of cancer. Zhang et al. found that ATP2A1 expression was elevated in CRC, and patients with high ATP2A1 levels had significantly poorer OS compared to those with low levels (35). CRYAB, a member of the small heat shock protein superfamily, was found to be elevated in CRC and play a significant role in promoting the invasion and metastasis of CRC through the process of epithelial-mesenchymal transition (EMT) (36). Liu et al. demonstrated that ELFN2, which is upregulated due to promoter hypomethylation in astrocytoma patients, is significantly associated with poor prognosis (37). Fan et al. demonstrated that MAT1A and PHB1 formed a complex and cooperated to defend the liver against metastasis of cancers (38). Wu et al. reported that ORC1 is overexpressed in the majority of cancers (39). Li et al. reported that POU4F1 is upregulated in colon cancer and facilitates the proliferation and migration of colon cancer cells (40). Although research has explored the role of these genes, the correlation between these eight genes and CRC progression remains to be elucidated. In the present study, we identified ARL6IP4 as the gene with the highest coefficient in the multivariate Cox regression analysis for further experimental validation. ARL6IP4, also referred to as SR-25, is recognized as a nuclear protein and may play a role in RNA splicing (41). However, the expression and role of ARL6IP4 in cancer, particularly in CRC, remain rarely explored. Our qRT–PCR analysis revealed that ARL6IP4 was significantly upregulated in the majority of paired CRC samples. More importantly, experiments conducted on live CRC cells have demonstrated that ARL6IP4 undergoes LLPS. In the future, we aim to investigate the biological functions and underlying mechanisms of ARL6IP4 in CRC.

The tumor microenvironment (TME) comprises a variety of immune cell types, cancer-associated fibroblasts, endothelial cells, pericytes, and numerous other tissue-resident cell populations (42). Some studies have indicated that the TME plays a significant role in cancer progression, such as metastasis, angiogenesis, and the regulation of immune infiltration and response. These factors subsequently influence strategies for cancer treatment and prognosis (43). Furthermore, the TME may be affected by oncogene-driven alterations in tumor cell metabolism that suppress immune responses and create barriers to effective cancer therapies (44). Emerging evidence underscored that LLPS not only facilitated the assembly of membraneless organelles within the TME (45), but was also intricately linked to immune cell functionality in cancer, playing a pivotal role in tumor immunosuppression (46). In our study, the low-risk group exhibited significantly higher levels of infiltration by anti-tumor immune cells, including M1 macrophages. M1 macrophages play a crucial role in activating the immune response and inhibiting tumor progression (47). Furthermore, the levels of memory B cells and Tregs in the high-risk group were significantly higher than those observed in the low-risk group. Tregs are recognized as a primary source of immunosuppressive cell types and elevated levels of Tregs are correlated with poorer prognoses in patients across various cancer types (48, 49). Research has pointed out that Treg cells contribute to tumorigenesis and development through the inhibition of adaptive anti - tumor immunity, which represents the key mechanism underlying tumor immune escape (50). This could explain the high-risk group’s tendency for immune evasion due to elevated TIDE scores. Our findings suggested that this high-risk group had established an immune-evasive microenvironment, which subsequently undermined antitumor immune responses and contributed to unfavorable clinical outcomes.

Current therapeutic approaches for CRC encompass surgical intervention, which serves as the foundation of curative treatment, alongside adjunctive therapies such as chemotherapy and targeted therapy (51, 52). Chemotherapy with or without targeted therapy is usually used in clinical practice, but how to judge whether patients, especially those with advanced CRC, are sensitive to the selected therapeutic drugs is still a major challenge in clinical medicine. Recent studies suggested that phase-separated condensates regulate drug distribution and concentration, significantly impacting drug efficacy, and this mechanism may be associated with drug resistance in cancer therapy (53). In colon cancer, research indicated that SENP1 diminished the phase separation of RNF168, thereby enhancing DNA damage repair and contributing to drug resistance (54). Our findings showed that the risk signature could differentiate patients’ sensitivity to drug use, including common chemotherapeutic drugs like oxaliplatin, 5-FU. The standard adjunctive treatment for patients with CRC typically involves the use of fluoropyrimidine agents, such as 5-FU or capecitabine, either as monotherapy or in combination with oxaliplatin (55). These results indicated that our risk signature could not only predict the prognosis of CRC patients, but also predict the sensitivity of patients to drugs.

Our analyses indicated that the risk signature we developed can effectively predict prognosis, immune landscape, and drug sensitivity in CRC patients. However, this study still has limitations. First, the data were obtained from public databases and retrospective. Therefore, it is essential to obtain more large-scale and prospective real-world cohort to further validate the authenticity and robustness of the signature. Additionally, while we validated the expression level and phase separation capability of ARL6IP4 through qRT-PCR and phase separation assays, the role of ARL6IP4 phase separation in CRC still requires further investigation through both *in vitro* and *in vivo*. Moreover, our study primarily evaluated the prognostic significance and clinical relevance of the signature; however, the molecular mechanisms associated with the eight genes necessitate further investigation. Finally, the analyses related to immunotherapy and drug sensitivity in this study necessitate clinical validation to further establish their clinical significance.

## Conclusion

In summary, we constructed a risk signature using eight LRGs in CRC patients, which outperforms traditional TNM staging system in predicting patient outcomes. This signature can also assess immunotherapeutic responses and drug sensitivity, which facilitates more precise personalized treatment strategies for CRC patients. Additionally, the expression and phase separation ability of ARL6IP4 were confirmed through experiments. However, further prospective real-world CRC cohort is need to verify the efficiency of the risk signature. Moreover, further *in vitro* and *in vivo* experiments are required to explore the expression level, biological functions, and underlying mechanisms of ARL6IP4 and the other seven genes in CRC. Taken together, our study offers new insights into CRC prognosis prediction and may enhance clinical treatment.

## Data Availability

Publicly available datasets were analyzed in this study. This data can be found here: GEO (https://www.ncbi.nlm.nih.gov/geo/, accession numbers: GSE39582) and TCGA (https://portal.gdc.cancer.gov/, accession numbers: TCGA-CRC).
