# Supplementary material for: A novel liquid-liquid phase separation related gene signature including ARL6IP4 predicts prognosis and immune landscape in colorectal cancer
Source: Front Immunol. 2026 Jan 6;16:1694377. doi: 10.3389/fimmu.2025.1694377 (PMC12816288; doi:10.3389/fimmu.2025.1694377)
Supplement: Supplementary file 1 [file DataSheet1.docx]

**Supplementary Table S1** Clinical characteristics of training and testing cohorts in TCGA-CRC database.

| Variables | Entire | Test | Train | Pvalue |
| --- | --- | --- | --- | --- |
| Age |  |  |  |  |
| <=65 | 236(43.54%) | 122(45.02%) | 114(42.07%) | 0.5442 |
| >65 | 306(56.46%) | 149(54.98%) | 157(57.93%) |  |
| Gender |  |  |  |  |
| FEMALE | 255(47.05%) | 124(45.76%) | 131(48.34%) | 0.6056 |
| MALE | 287(52.95%) | 147(54.24%) | 140(51.66%) |  |
| Stage |  |  |  |  |
| Stage I | 93(17.16%) | 47(17.34%) | 46(16.97%) | 0.3325 |
| Stage II | 208(38.38%) | 111(40.96%) | 97(35.79%) |  |
| Stage III | 148(27.31%) | 66(24.35%) | 82(30.26%) |  |
| Stage IV | 78(14.39%) | 43(15.87%) | 35(12.92%) |  |
| unknow | 15(2.77%) | 4(1.48%) | 11(4.06%) |  |
| T stage |  |  |  |  |
| T1 | 15(2.77%) | 7(2.58%) | 8(2.95%) | 0.8357 |
| T2 | 93(17.16%) | 44(16.24%) | 49(18.08%) |  |
| T3 | 370(68.27%) | 188(69.37%) | 182(67.16%) |  |
| T4 | 63(11.62%) | 31(11.44%) | 32(11.81%) |  |
| Tis | 1(0.18%) | 1(0.37%) | 0(0%) |  |
| M stage |  |  |  |  |
| M0 | 402(74.17%) | 200(73.8%) | 202(74.54%) | 0.3924 |
| M1 | 77(14.21%) | 43(15.87%) | 34(12.55%) |  |
| unknow | 63(11.62%) | 28(10.33%) | 35(12.92%) |  |
| N stage |  |  |  |  |
| N0 | 318(58.67%) | 163(60.15%) | 155(57.2%) | 0.5135 |
| N1 | 129(23.8%) | 66(24.35%) | 63(23.25%) |  |
| N2 | 94(17.34%) | 42(15.5%) | 52(19.19%) |  |
| unknow | 1(0.18%) | 0(0%) | 1(0.37%) |  |

**
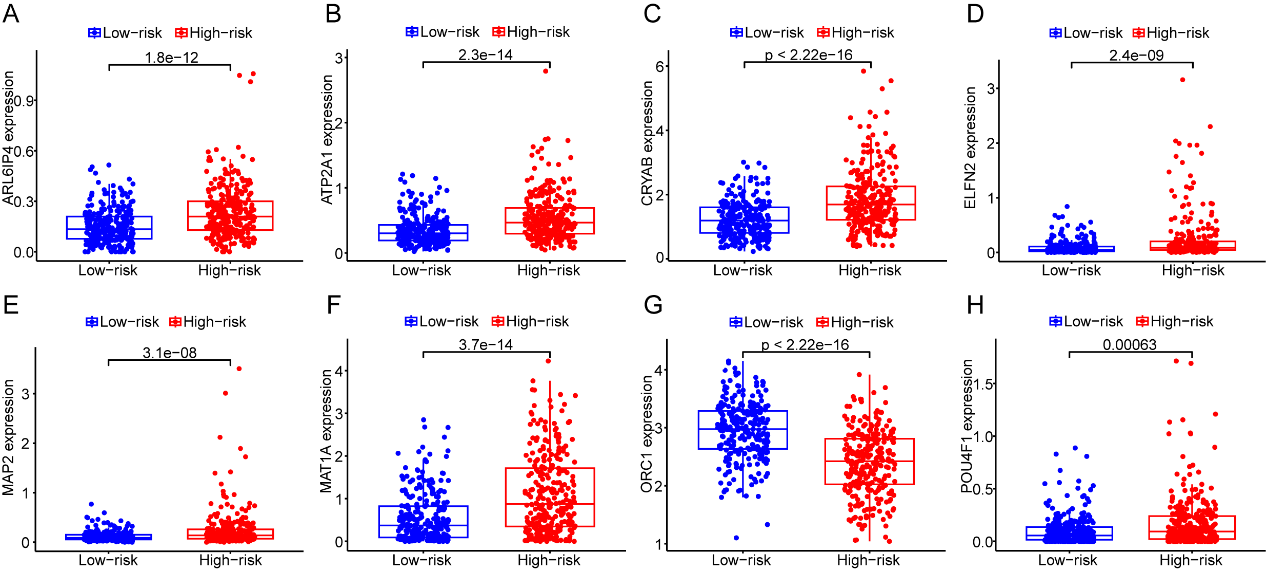
**

**Supplementary Figure S1** Differential expression analysis of the eight signature genes between low-risk and high-risk groups. (**A**) ARL6IP4, (**B**) ATP2A1, (**C**) CRYAB, (**D**) ELFN2, (**E**) MAP2, (**F**) MAT1A, (**G**) ORC1, and (**H**) POU4F1.

**
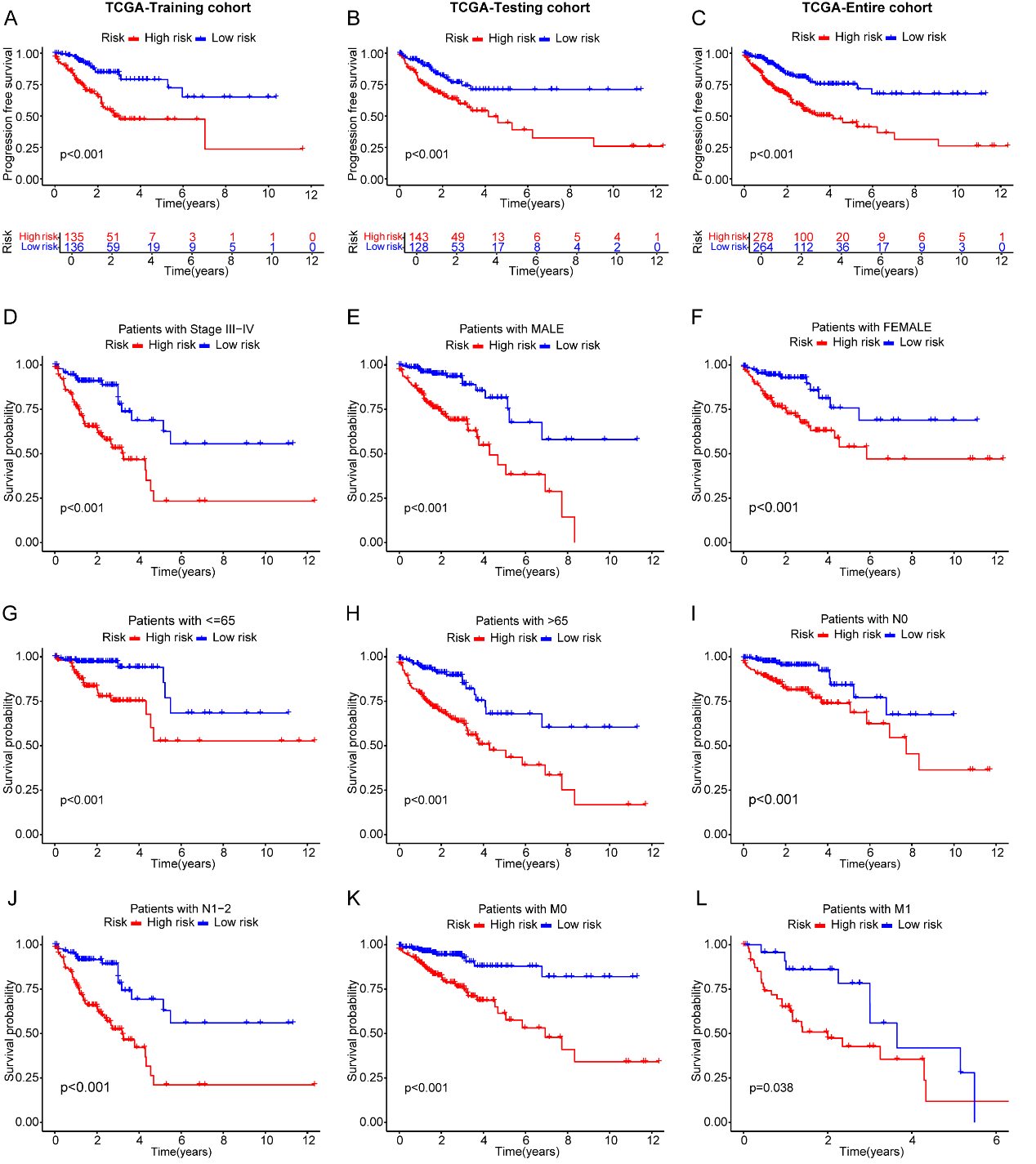
**

**Supplementary Figure S2** Prognostic verification of the risk signature**.** (**A-C**) K-M curves illustrating PFS for the two risk groups within the TCGA-CRC across the training (A), testing (B), and entire (C) cohorts. (**D-L**) K-M curves illustrate prognosis across various subgroups, including tumor stages III–IV (D), male (E) and female (F), aged ≤65 years (G) and >65 years (H), N0 (I), N1-2 (J), M0 (K), and M1 (L) classifications.

**
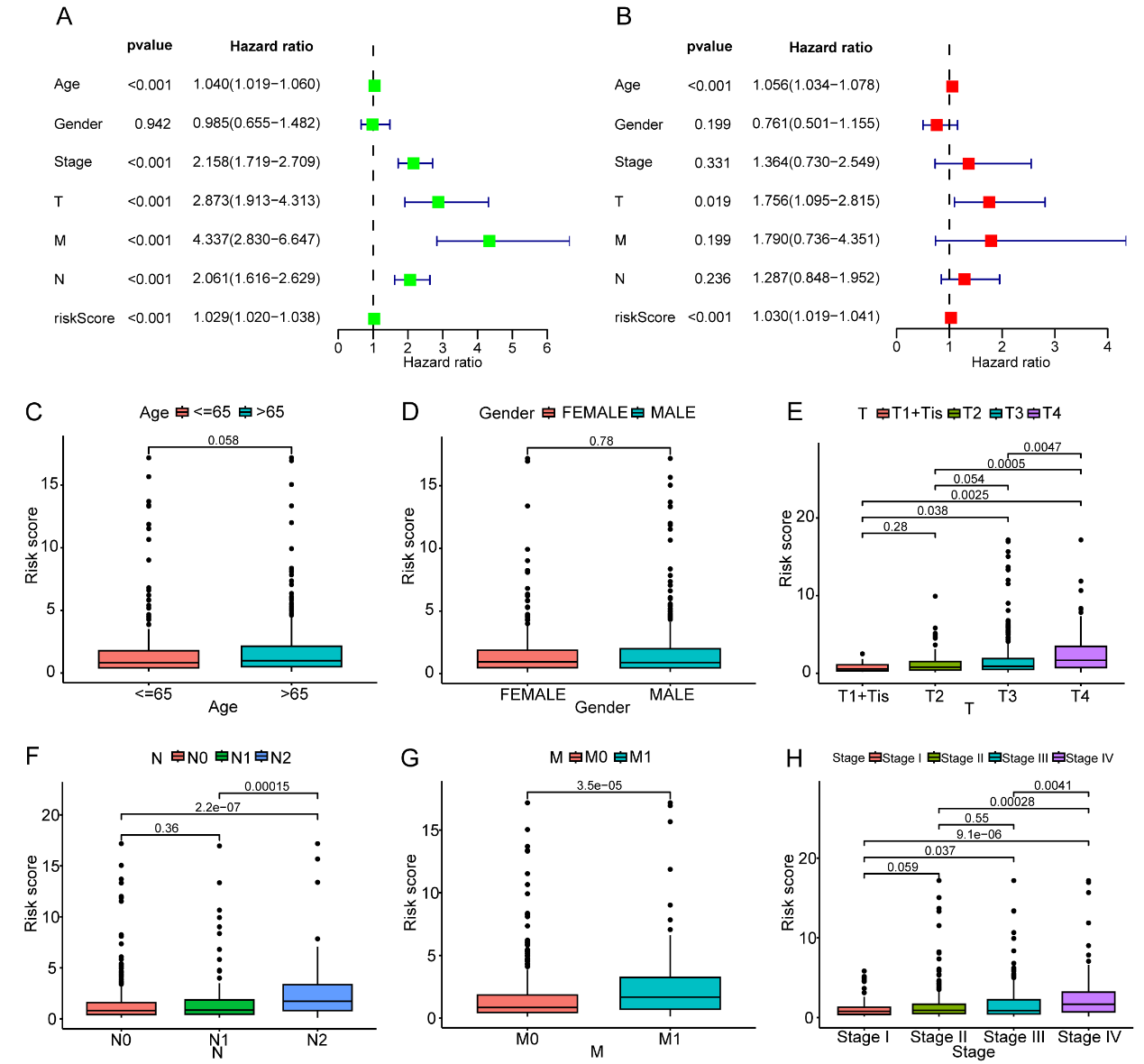
Supplementary Figure S3** Independent prognostic significance and clinicopathological features of the risk signature. (**A, B**) Univariate and multivariate Cox regression analyses showing the prognostic value of the risk signature and clinicopathological features. (**C-H**) The histograms showing the differences of the risk score in groups classified by age (C), gender (D), T stage (E), N stage (F), M stage (G), and tumor stage (H).

**
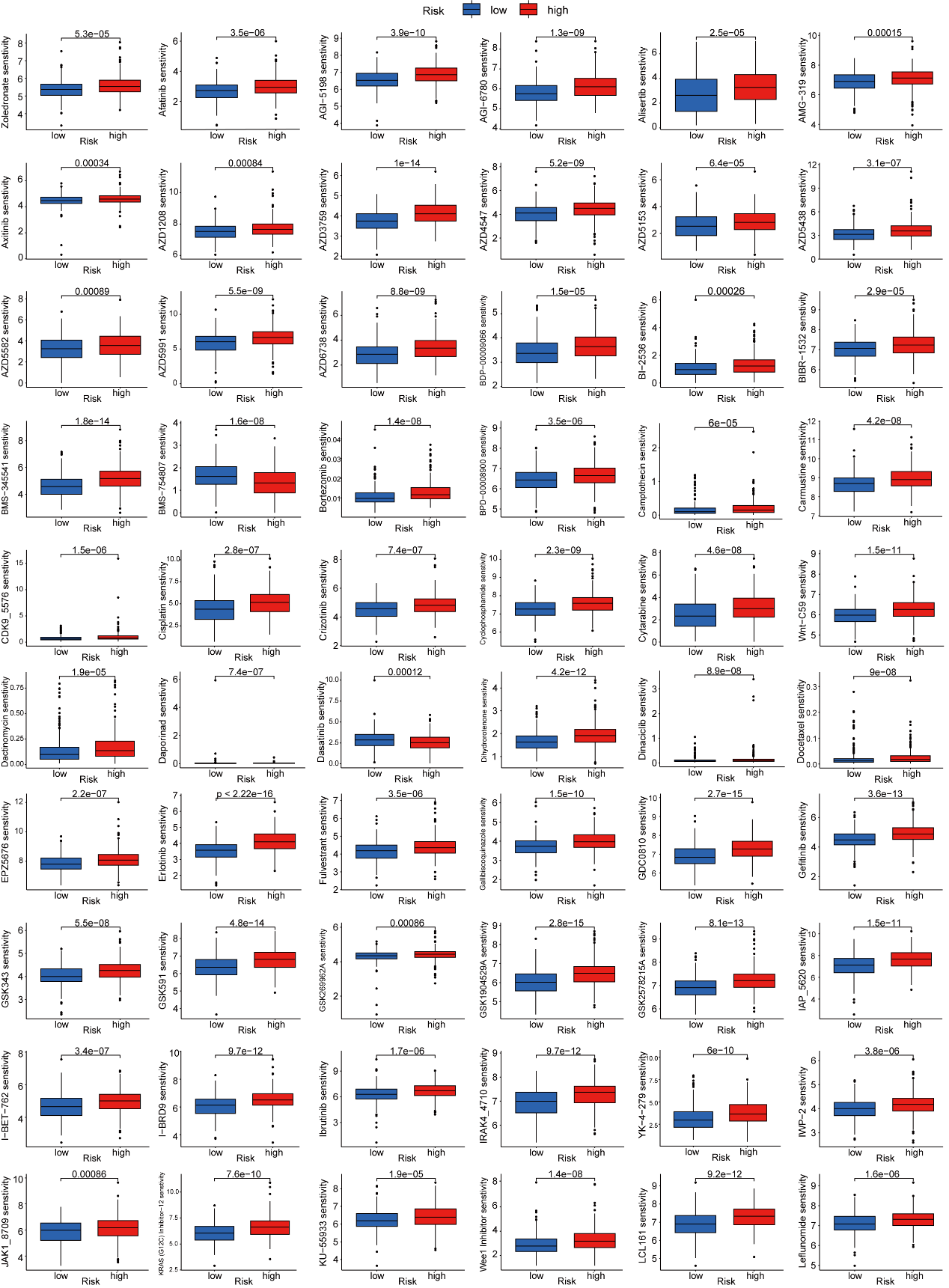
**

**Supplementary Figure S4** Analysis of differential drug sensitivity in the low-risk and high-risk groups.

**
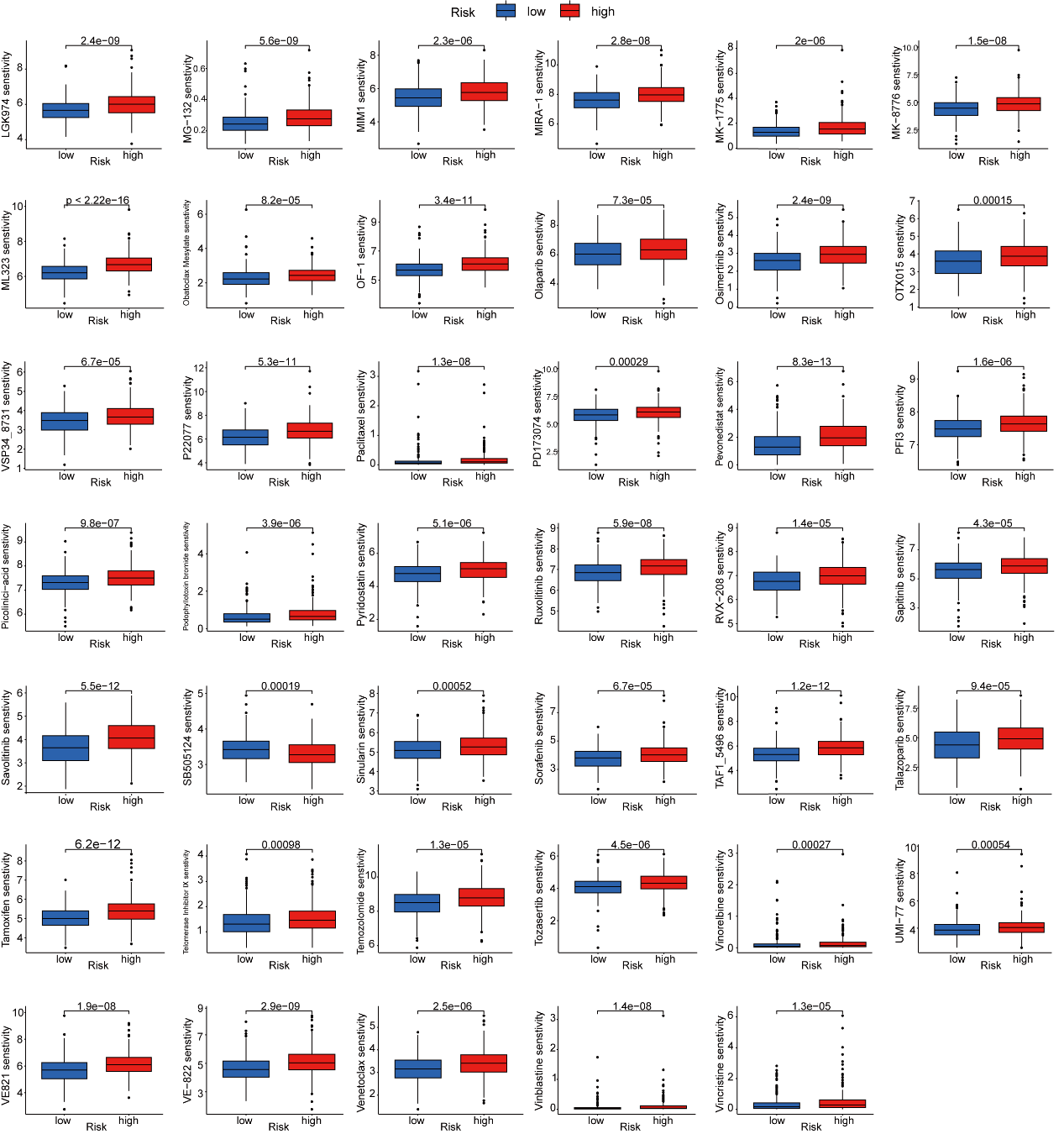
**

**Supplementary Figure S5** Analysis of differential drug sensitivity in the low-risk and high-risk groups.


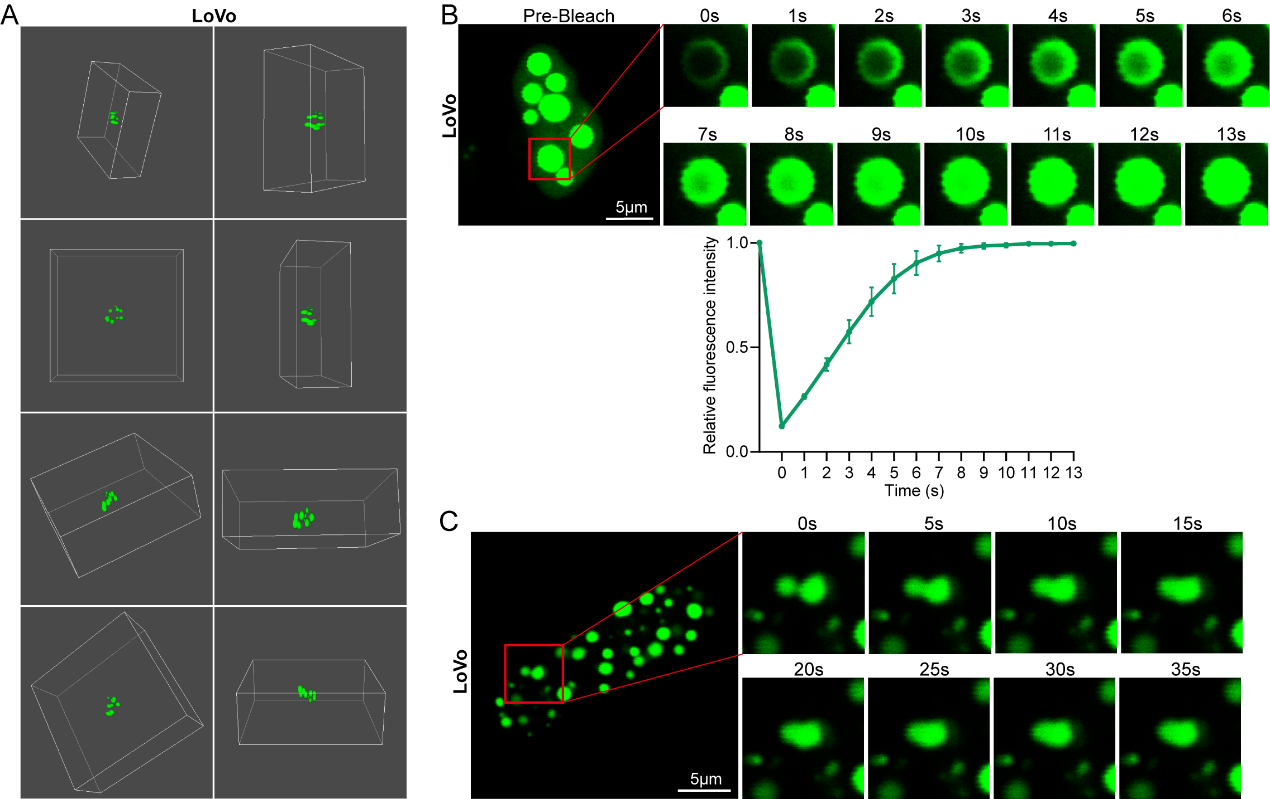


**Supplementary Figure S6** Phase separation capability of ARL6IP4 in CRC. **(A)** 3D-reconstructed images of live LoVo cells transfected with EGFP-ARL6IP4 plasmid were acquired using a confocal laser scanning microscope. **(B)** Images (top) and quantitative analysis (bottom) of EGFP-ARL6IP4 FRAP were presented in LoVo cells. Data was expressed as Mean ± SE, with n = 3 independent biological replicates. **(C)** Two EGFP-ARL6IP4 droplets in LoVo cells underwent fusion to create a larger droplet.
